# Differential effects of antidepressants escitalopram versus lithium on Gs alpha membrane relocalization

**DOI:** 10.1186/s12868-015-0178-y

**Published:** 2015-07-11

**Authors:** Robert J Donati, Jeffrey Schappi, Andrew H Czysz, Alexander Jackson, Mark M Rasenick

**Affiliations:** Departments of Physiology and Biophysics, College of Medicine, University of Illinois at Chicago, Chicago, IL 60612-7342 USA; The Psychiatric Institute, College of Medicine, University of Illinois at Chicago, Chicago, IL 60612-7342 USA; Basic and Health Science Department, Illinois College of Optometry, Chicago, IL 60616 USA

## Abstract

**Background:**

Plasma membrane localization can play a significant role in the ultimate function of certain proteins. Specific membrane domains like lipid rafts have been shown to be inhibitory domains to a number of signaling proteins, including Gsα, and chronic antidepressant treatment facilitates Gs signaling by removing Gsα form lipid rafts. The intent of this study is to compare the effects of the selective serotnin reuptake inhibitor, escitalopram, with that of the mood stabilizing drug, lithium.

**Results:**

There are a number of mechanisms of action proposed for lithium as a mood stabilizing agent, but the interactions between G proteins (particularly Gs) and mood stabilizing drugs are not well explored. Of particular interest was the possibility that there was some effect of mood stabilizers on the association between Gsα and cholesterol-rich membrane microdomains (lipid rafts), similar to that seen with long-term antidepressant treatment. This was examined by biochemical and imaging (fluorescence recovery after photobleaching: FRAP) approaches. Results indicate that escitalopram was effective at liberating Gsα from lipid rafts while lithium was not.

**Conclusions:**

There are a number of drug treatments for mood disorders and yet there is no unifying hypothesis for a cellular or molecular basis of action. It is evident that there may in fact not be a single mechanism, but rather a number of different mechanisms that converge at a common point. The results of this study indicate that the mood stabilizing agent, lithium, and the selective serotonin reuptake inhibitor, escitalopram, act on their cellular targets through mutually exclusive pathways. These results also validate the hypothesis that translocation of Gsα from lipid rafts could serve as a biosignature for antidepressant action.

## Background

Despite several decades of research, no unifying hypothesis for a molecular or cellular basis of action for antidepressant drugs or mood-stabilizing drugs has emerged. Several studies in both human and animal tissue suggest that there is lower cAMP production in depressed individuals (see reviews [1, 2]) while a recent report using PET imaging suggests a global decrease of cAMP in brains from depressed subjects [3]. The reviews above also suggest that effective antidepressant treatment increases adenylyl cyclase (AC) activity. It has also been observed that effective antidepressants induce movement of Gsα out of lipid rafts, thus increasing the association between Gsα and AC, elevating AC activity, and increasing cAMP [4–6]. Gsα from C6 rat glioma cells migrates from a Triton X-100 (TX-100) resistant lipid raft containing membrane domain to a TX-100 soluble non-lipid raft membrane domains in response to chronic antidepressant treatment [4, 7] revealing Gsα as a preferential target for antidepressant action [4]. Taken together, these studies suggest that the lipid environment of the G protein may play an important role in its localization and function, and that chronic antidepressant treatment alters the membrane localization of Gsα, augmenting coupling to AC [7].

Human post-mortem data reveals that depressed subjects have more Gsα in the TX-100 resistant (raft enriched domains) compared to control subjects [8]. These results suggest that Gsα is less available for AC signaling in the depressed brain and is consistent with the observation that a therapeutic effect of antidepressants may be to move Gsα out of TX-100 resistant regions of the membrane and into a membrane domain where it is more available to interact with AC. Additionally, Gsα-mediated AC activity is significantly lower in platelets from depressed subjects [9, 10]. Similar to results seen in cell culture these studies suggest that during depression, Gsα is sequestered in lipid raft-like domains and antidepressant treatment liberates the Gsα from these inhibitory domains allowing it to more freely couple to AC. Furthermore, antidepressant drugs concentrate in raft-like plasma membrane domains [11]. Thus, antidepressants may exert their observed effects on cAMP signaling by liberating Gsα from lipid rafts, where it accumulates during the course of depression [8, 12].

Lithium continues to be the primary pharmacological treatment for bipolar disorder. It has been shown that lithium and other mood stabilizers inhibit AC activity [13, 14] with lithium having a preference for Types V and VII AC [15]. There are numerous hypotheses on the mechanism of action of lithium (review by Marmol [16]) with one of the more current mechanisms involving inhibition of GSK-3 (review by Machado-Vieira et al. [17]). The involvement of serotonin and dopamine regulation of GSK-3 activity in the action of psychotropic drugs has also been recently reviewed [18–21]. Previous studies have shown that the effects of lithium in the treatment of affective disorders may be due to inhibition/decrease of the G-protein Giα activity and an increase in AC types I and II activity [22].

Currently, it is not possible to directly and noninvasively monitor the activity of AC or the partitioning of Gsα. However, if a biomarker can be identified for the process of making Gsα available to complex with AC, it would then be possible to quantify whether a given drug treatment is altering Gsα-AC coupling in a shorter time period, weeks vs months, predicting a likely therapeutic success. It is our current hypothesis that chronic antidepressant treatment, but not chronic treatment with other classes of drugs used to treat mood disorders, may alter lipid raft composition (lipids, proteins or both) so that the anchor for Gsα is lost and Gsα moves into non-raft fractions, increasing its availability to activate AC [12]. Escitalopram, the therapeutically active *S*-enantiomer of citalopram, liberated Gsα from detergent resistant membranes of chronically treated C6 glioma cells, while *R*-citalopram or acute drug treatment had no effect [23].

The results of the current study demonstrate that escitalopram facilitates the release of Gsα, but not Giα, from detergent resistant membrane domains while lithium and valproic acid do not have this effect. In fact, lithium and valproic acid may actually increase the movement of Gsα into these detergent resistant membrane domains.

## Results

### Effect of antidepressant vs lithium on Gsα TX-100 resistant membrane localization

**Figure 1:**
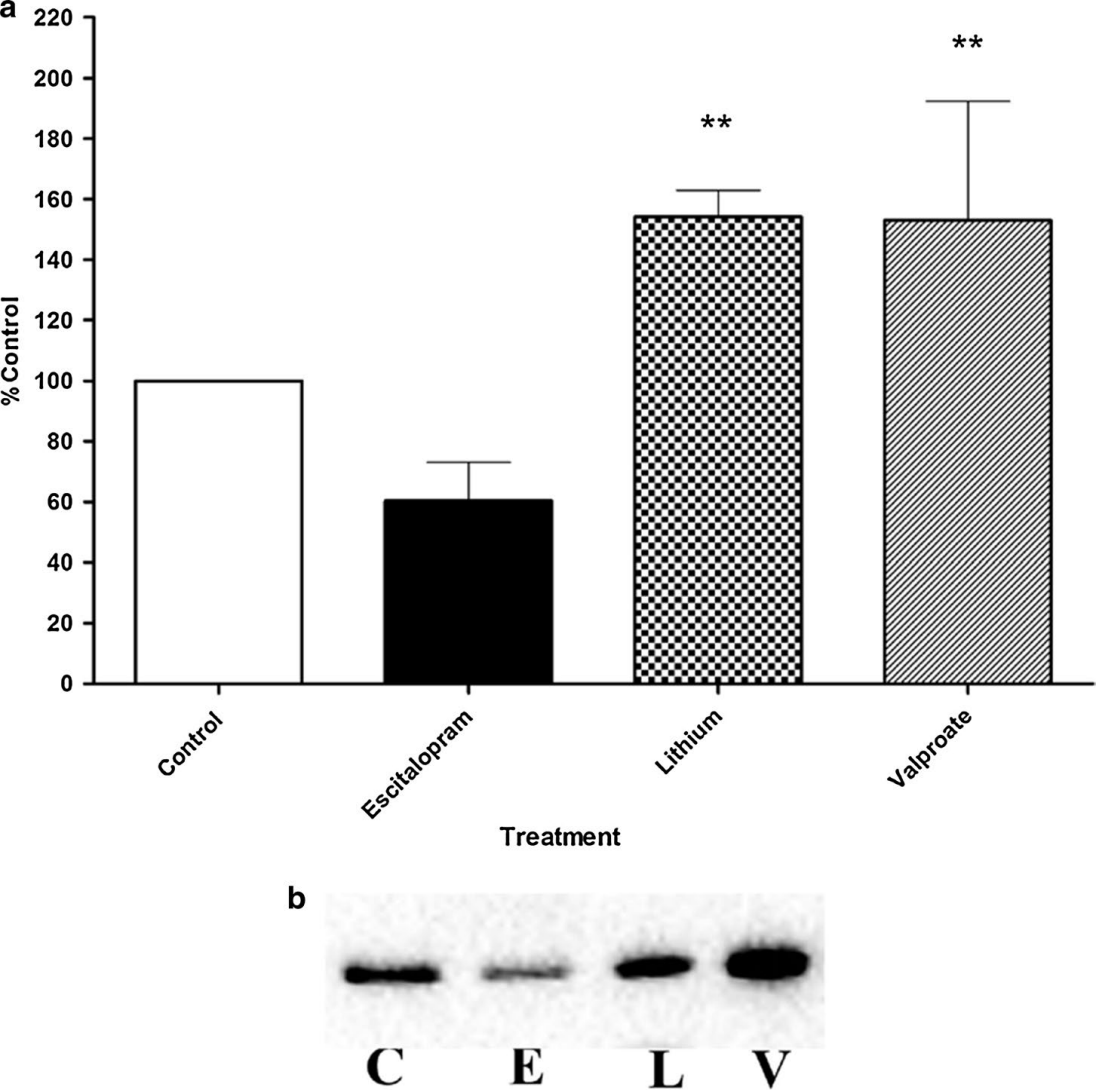
Gsα is removed from lipid rafts subsequent to antidepressant treatment while lithium has the opposite effect. Cells were treated as indicated in “Methods” and lipid rafts were isolated using sucrose gradient flotation. The amount of Gsα was determined by Western blot. **a** The *figure* shows the percentage of change in Gsα protein compared to control in the lipid raft membrane fractions (n = 7 for control, escitalopram and lithium; n = 5 for valproic acid). Data were analyzed by one-way ANOVA (p < 0.0006) followed by Tukey’s multiple comparison test for post hoc comparison of means. Data are represented as mean ± SEM (**, p < 0.005 compared to escitalopram). **b** A representative immunoblot of lipid raft Gsα from the various treatment paradigms as explained in “Methods”. *C* control, *E* escitalopram, *L* lithium, *V* valproic acid.

Chronic antidepressant treatment has been shown to decrease the localization of Gsα in detergent resistant membrane domains (lipid rafts), both in cerebral cortex membranes from rats and in C6 glioma cells [4, 7, 23]. Despite its structural similarity to tricyclic antidepressants, chlorpromazine did not have this effect [7, 25]. To test whether two of the more common mood stabilizing drugs, lithium and valproic acid, had similar effects as antidepressants, C6 cells were exposed to escitalopram, lithium, or valproic acid. Extraction of the membranes with TX-100 followed by sucrose density gradient centrifugation allows for the purification of signaling molecules within detergent resistant membranes [24]. The results show that while escitalopram reduced the amount of Gsα in the lipid raft compared with drug-free control cells, lithium and valproic acid had the opposite effect (Figure 1). There is translocation of Gsα out of lipid raft domains caused by the antidepressant escitalopram and a shift of Gsα into the lipid raft domains by the mood stabilizing agents, lithium and valproic acid compared to control. Additionally, there is a significant difference compared to escitalopram treatment (p < 0.005 for valproic acid and lithium).This experiment demonstrated that an antidepressant stimulated the movement of Gsα out of lipid rafts, while the two mood stabilizers had the opposite effect.

### Effect of antidepressant vs lithium on Giα TX-100 resistant membrane localization

**Figure 2:**
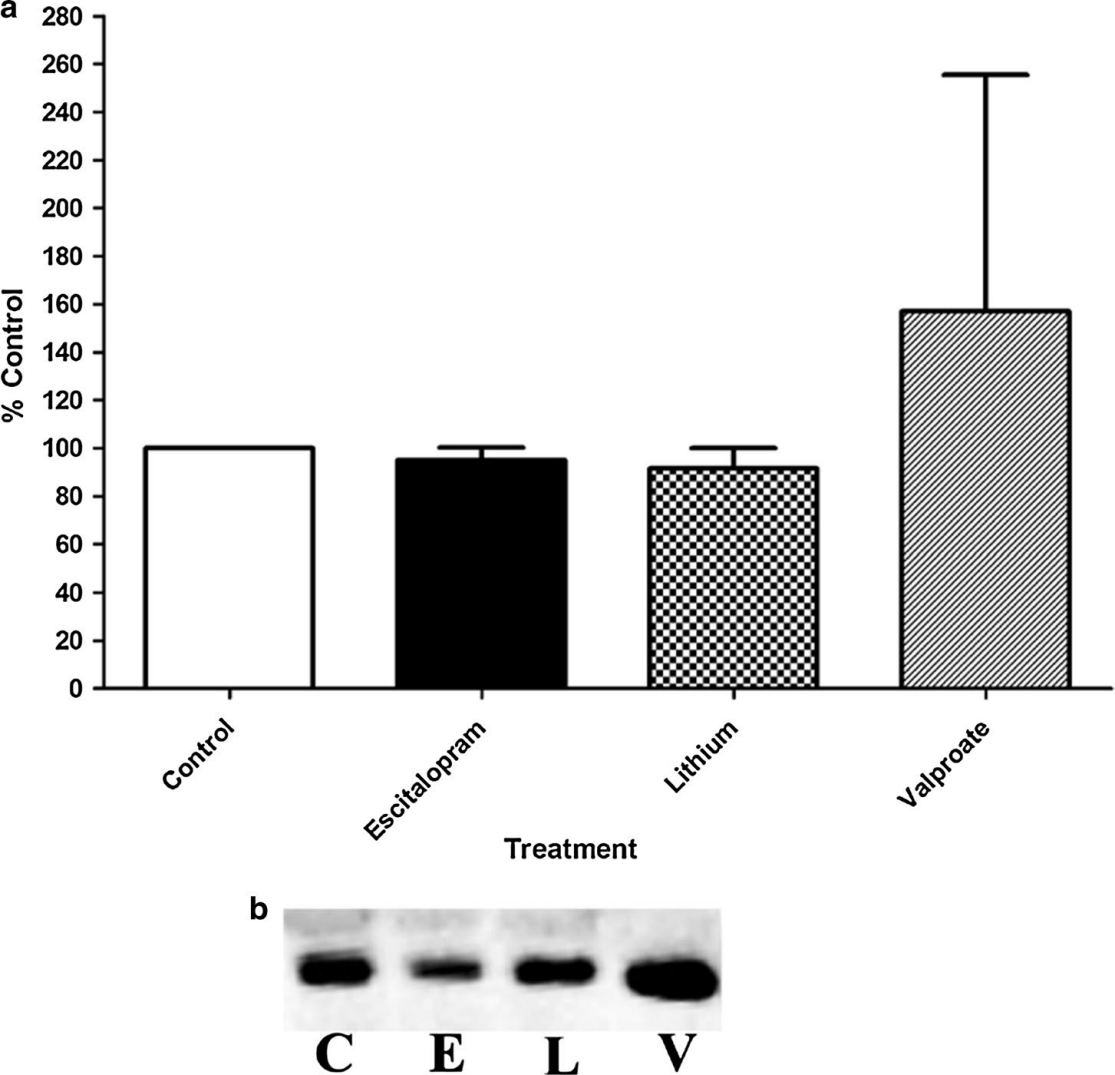
Giα membrane compartmentalization is not altered by chronic antidepressant or lithium treatment. Purified lipid rafts isolated using sucrose gradient flotation revealed that neither chronic antidepressant treatment nor chronic lithium treatment move Giα out of lipid rafts. **a** The *figure* shows the percentage of change in Giα protein in the lipid raft membrane fractions compared to control (n = 4 for control, escitalopram, and lithium; n = 3 for valproate). Data were analyzed by one-way ANOVA followed by Tukey’s multiple comparison test for post hoc comparison of means. Data are represented as mean ± SEM **b** A representative immunoblot of lipid raft Gsα from the various treatment paradigms as explained in “Methods”. *C* control, *E* escitalopram, *L* lithium, *V* valproic acid.

Previous studies have shown that, while a number of different antidepressants alter the association of Gsα with lipid rafts, the localization of Giα remains unaffected [4, 7, 25]. This suggested that the antidepressants were not altering lipid rafts, but were altering the anchoring of Gsα to those rafts. Figure 2 demonstrates that neither escitalopram nor lithium alters the membrane localization of Giα in C6 glioma cells (Figure 2). While valproic acid appears to increase the amount of Giα in the lipid rafts, there is a much greater variability between trials compared to escitalopram and lithium treatment. Congruent with this result and the results of the study mentioned above, it appears that enhancing Gsα mobility out of lipid raft domains may in fact, be a hallmark of antidepressant action.

### Effect of antidepressant vs lithium on Gsα and Giα membrane mobility

**Figure 3:**
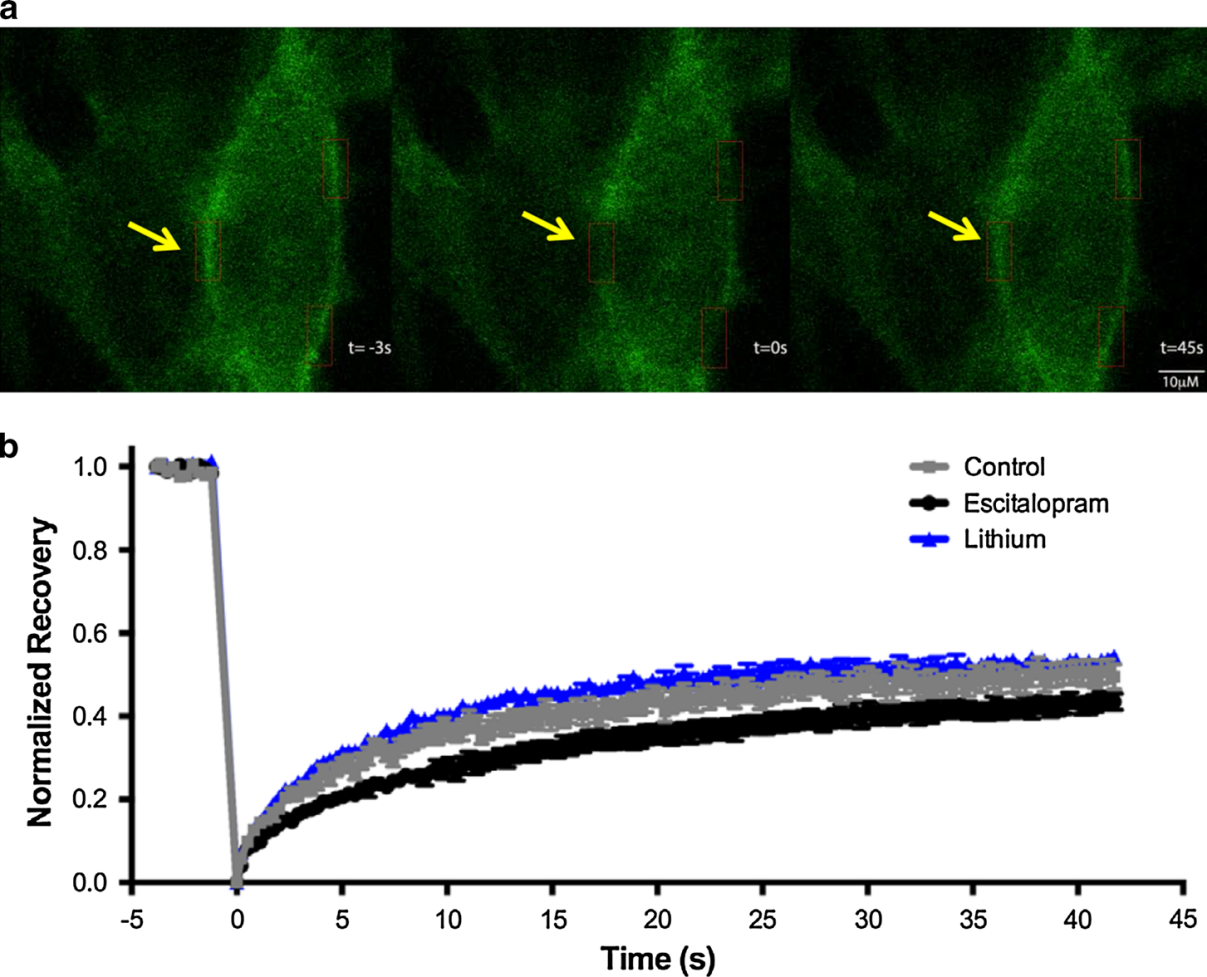
FRAP suggests increased release of GFP-Gsα from lipid rafts after chronic antidepressant but not lithium treatment. **a** Typical course of photobleaching with representative images of cell before photobleaching (t = −3 s), immediately after photobleaching (t = 0 s), and after maximal recovery of fluorescence (t = 45 s). **b** Demonstration of typical fluorescence recovery after photobleaching in cells treated with escitalopram 10 µM or LiCl 3 mM for 72 h as described in “Methods”. *Yellow arrows* indicate area of bleach and recovery.

**Figure 4:**
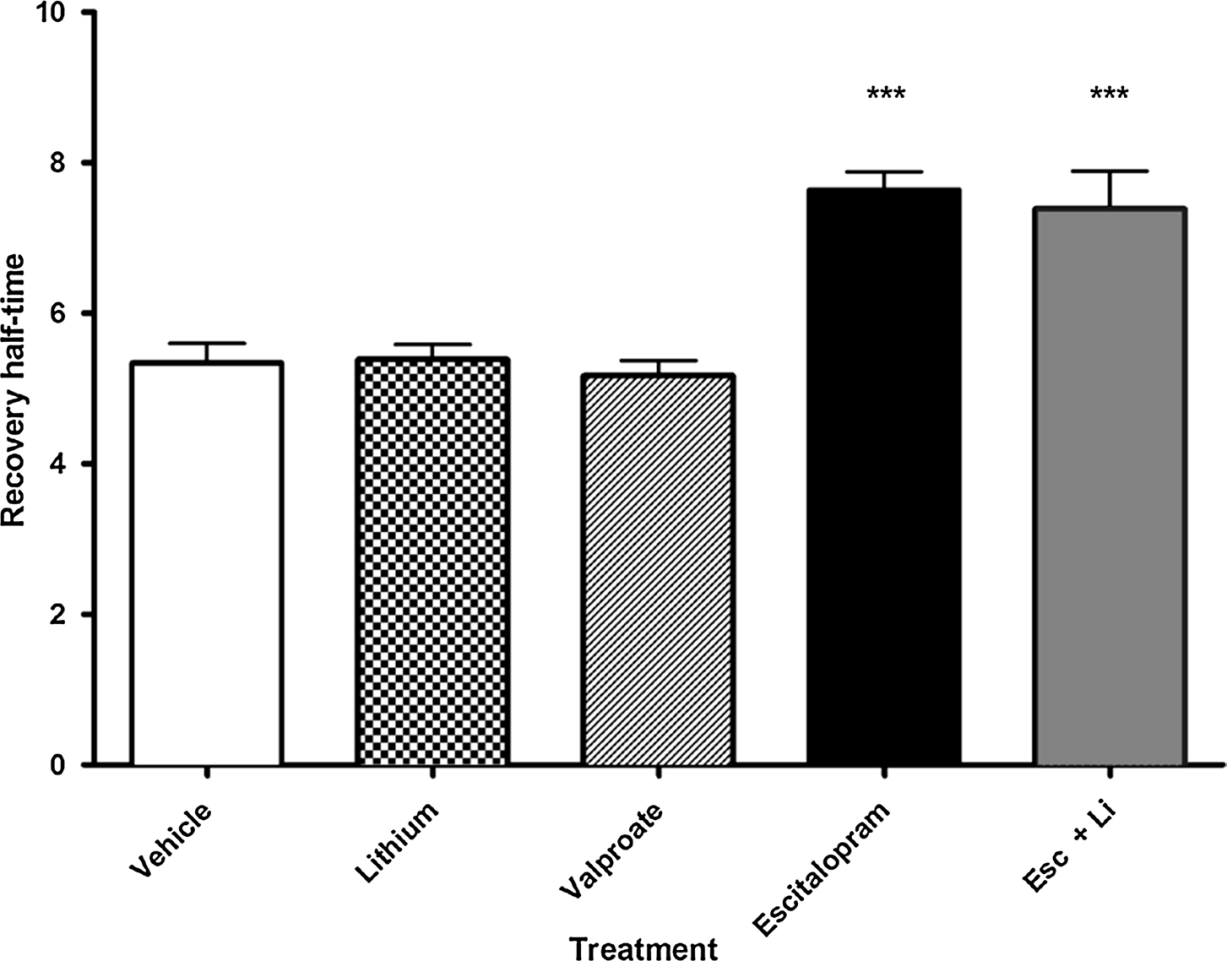
FRAP suggests that lithium combined with antidepressant does not reverse the effect of antidepressant GFP-Gsα was stably expressed in a C6 glioma cell line. Half-time to recovery of GFP-Gsα is increased after chronic escitalopram treatment, suggesting increased coupling with its effector, adenylyl cyclase. Lithium combined with escitalopram does not affect this value, suggesting an alternate locus of action for lithium. GFP-Gsα recovery is not altered by lithium or valproic acid treatment, suggesting unaltered coupling of GFP-Gsα with adenylyl cyclase. Sample size represents the number of cells assayed, with a minimum of 21 and a maximum of 101 cells assayed per experiment. Data were analyzed by one-way ANOVA (p < 0.0001) followed by Tukey multiple comparison test for post hoc comparisons of means. Data are represented as mean ± SEM (***, p < 0.0005 compared to vehicle).

**Figure 5:**
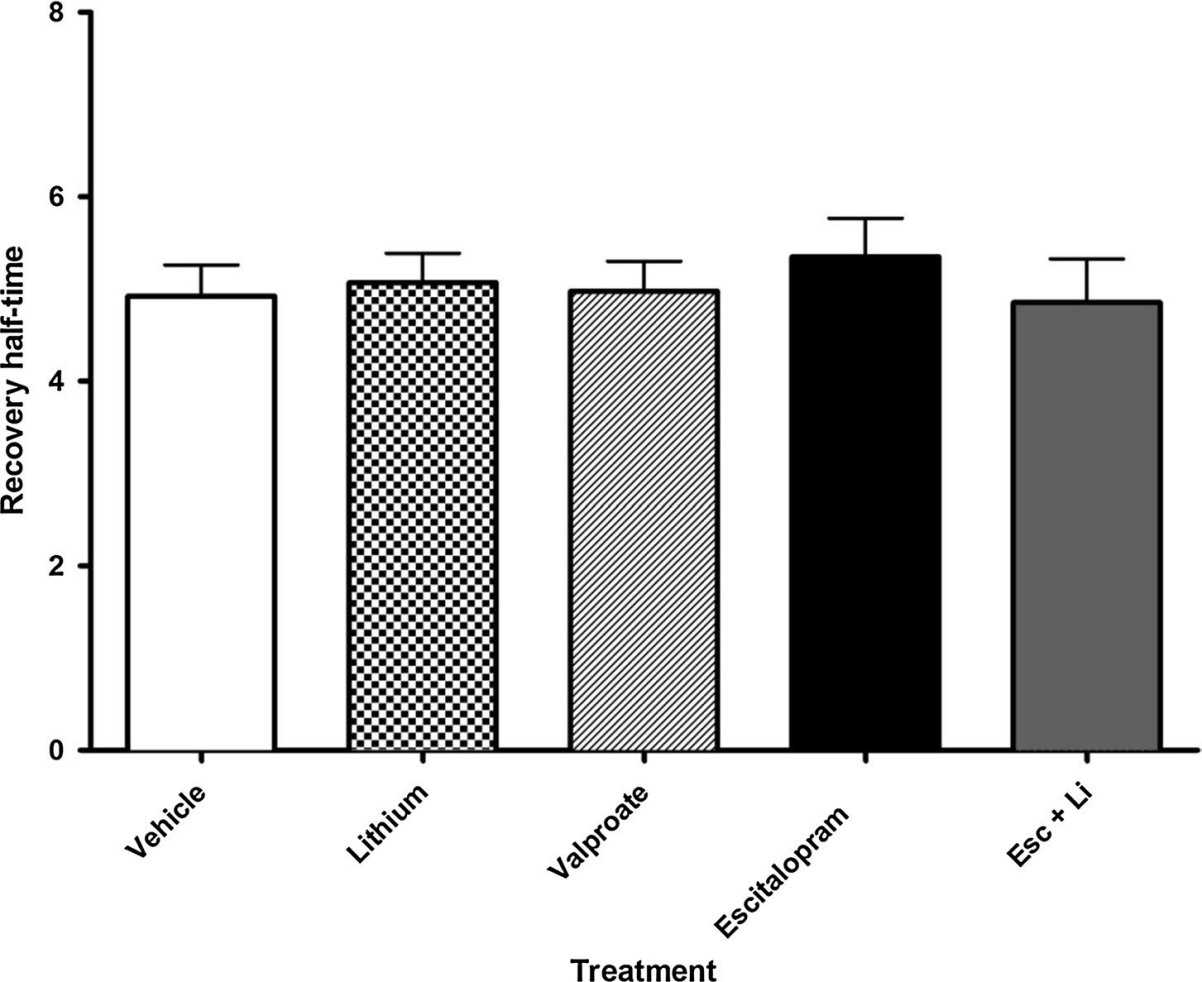
GFP-Giα FRAP is unaffected by chronic antidepressant or mood stabilizer treatment. Giα-GFP was stably expressed in a C6 glioma cell line. Half-time to recovery of Giα-GFP is not altered by chronic treatment with escitalopram, lithium, or valproic acid. This suggests that the change in membrane compartmentalization of Gsα is not due to direct effects of these agents on lipid rafts, but that the effects of antidepressants are specific to Gsα. Sample size represents the number of cells assayed, with a minimum of 26 and a maximum of 46 cells assayed per experiment. Data were analyzed by one-way ANOVA followed by Tukey multiple comparison test for post hoc comparisons of means. Data are represented as mean ± SEM.

Lateral mobility within the plasma membrane of Gsα as determined by fluorescence recovery after photobleaching (FRAP), changed following 1–3 days (depending on concentration) of antidepressant treatment [12]. Specifically, the half-time to recovery of GFP-Gsα is increased, likely due the increased association of Gsα, with the slow-moving adenylyl cyclase. In contrast, FRAP of GFP-Giα_1_ was unchanged after antidepressant treatment. A typical membrane bleaching and recovery of fluorescence is shown in Figure 3a. Escitalopram treated cells demonstrate a shorter total recovery (increased “immobile fraction”), perhaps because Gsα is bound to adenylyl cyclase (Figure 3b). Additionally, escitalopram treated cells have a less steep recovery curve than either control or lithium treated cells, which have similarly shaped curves. That is, they recover their fluorescence more slowly (increased half time of recovery). Figure 4 shows GFP-Gsα FRAP after 3 days of treatment with lithium chloride (3 mM), valproic acid (300 μM), escitalopram (10 μM), or escitalopram plus lithium combined (same doses as individual treatments with these compounds). While lithium and valproic acid treatment show no effect on Gsα membrane mobility compared to vehicle, treatment with escitalopram or escitalopram plus lithium shows the characteristic slowing of Gsα mobility as demonstrated by an increase in recovery half-time compared to vehicle (p < 0.0005 for escitalopram and p < 0.0005 for escitalopram plus lithium). In contrast, FRAP of GFP-Giα_1_ is unaltered by the aforementioned treatments (Figure 5), suggesting again that the effects of these drugs are not mediated through actions on Giα_1_.

## Discussion

While primary targets of many antidepressant drugs may be proteins mediating monoamine uptake or catabolism, there may also be postsynaptic actions, including increased cAMP production and a cascade of events resulting from sustained increase in cAMP or the activation of Gsα [1]. Recent results suggest that Gsα/adenylyl cyclase coupling may be one of the targets of antidepressant action, and that chronic treatment is required to observe this effect via altered membrane localization [10]. Previous studies demonstrated that chronic antidepressant treatment-induced increases in Gsα movement from a TX-100 insoluble lipid raft rich domain to a TX-100 soluble membrane domain with a concurrent increase in coupling to adenylyl cyclase [7]. These results are consistent with a study revealing that a number of antidepressant drugs concentrate in detergent insoluble membrane domains, like lipid rafts, subsequent to chronic treatment [11]. Preclinical, platelet, and postmortem brain data indicate that, in depression, the G-protein Gsα is more likely to reside in detergent insoluble lipid rafts [5, 8, 9]. There is evidence that other proteins potentially involved in a mechanism of depression also show altered lipid raft localization. SERT clustering in lymphocyte lipid rafts is altered in a rat model of depression, relative to controls. [26].

The model system used in these studies, C6 glioma cells, has been used for antidepressant studies by this and other labs [4, 7, 11, 12, 23, 25, 37]. Direct comparisons have demonstrated similar effects of prolonged drug treatment as seen in rats, except that 3 weeks in rats were identical to 3 days in cell culture [7]. While the presumption is that neurons are the dominant factor in both depression and antidepressant response, a role for glia (alone or in combination with neurons) is both likely and unresolved.

Chronic antidepressant treatment (in rats and cultured cells) moves Gsα out of TX-100 insoluble lipid raft rich domains and into a domain more amenable to an association with adenylyl cyclase [1, 4], this action may explain the sustained increases in cAMP signaling that accompany antidepressant treatment [2]. A previous study also provided evidence that Gsα signaling was inhibited in TX-100 insoluble lipid raft rich domains [24]. These data are consistent with the hypothesis that liberation of Gsα from these detergent insoluble inhibitory membrane domains by chronic treatment with antidepressants leads to increased coupling between signaling molecules in the detergent soluble non-raft membrane regions.

Lithium has been used as a treatment for bipolar disorder for over 50 years and has been effective in treating both acute manic and depressive symptoms, in addition to reducing their occurrence [27, 28]. A number of studies have demonstrated that lithium inhibits adenylyl cyclase activity [29–32] and more specifically type V adenylyl cyclase [13]. Additionally, one potential mechanism of the antidepressant action of lithium is via inhibition of GSK-3 [14, 33–35]. Recent evidence suggests that lithium has a membrane delimited dual function in inhibiting the activation of G-protein activated K+ channels [36]. This was suggested to be due to decreased affinity of the Gα subunit for the Gβγ subunit and diminished GDP-GTP exchange on the Gα subunit. What is unknown is whether chronic lithium treatment has any effect on the TX-100 insoluble lipid raft membrane localization of Gsα. In this vein, we set out to test whether lithium has the ability to alter Gsα localization in TX-100 insoluble lipid raft rich domains in an antidepressant manner. In addition, we used a structurally disparate mood stabilizer, valproic acid, to see if the effects were similar to lithium. In light of mood stabilizers’ effect on Gsα membrane disposition as determined by membrane fractionation and immunoblotting (Figure 1), these data are not sufficient to suggest that Gsα movement into lipid raft fractions and the decreased cellular production of cAMP associated with lithium or valproic acid treatment [13] are due to a decreased association of Gsα with adenylyl cyclase. Rather, it likely reflects a G protein-independent pathway of adenylyl cyclase regulation.

Curiously, while the above membrane fractionation data demonstrates movement of Gsα into lipid rafts upon treatment with lithium (Figure 1), we did not observe changes in membrane localization of Giα (Figure 2), nor FRAP recovery half-time of either GFP-Gsα or GFP-Giα (Figures 3, 4, 5). One interpretation of this is that the physical coupling between GFP-Gsα or GFP-Giα and adenylyl cyclase is not altered by lithium treatment, and that regulation of cellular cAMP levels by lithium is not effected by direct G protein regulation of adenylyl cyclase. The lack of effect by lithium on escitalopram-mediated Gsα FRAP (Figure 4) further suggests a different locus of action. These effects on Gsα raft domain localization may partially explain why lithium is not effective as a stand alone treatment for chronic depression.

The decreased mobility of GFP-Gsα subsequent to antidepressant treatment was initially surprising to us, as we had hypothesized the “liberation” of Gsα from lipid raft domains would produce the opposite effect, increased mobility of GFP-Gsα and faster recovery of fluorescence after photobleaching (i.e., decreased half-time to recovery). Instead, we found, consistently, that the movement of Gsα out of lipid rafts upon antidepressant treatment was associated with a slowing of GFP-Gsα mobility and an increase in half-time to recovery. We have proposed that this phenomenon is due to the increased association of Gsα, a smaller, peripheral membrane protein, with the larger, multi-transmembrane spanning adenylyl cyclase, which displays extremely slow lateral mobility and has been shown to increase physical association with Gsα subsequent to antidepressant treatment [37]. This “molecular signature” is typical of the many antidepressants tested by our lab (all major groups and several atypical compounds) [4, 7, 12, 23]. Other psychotropic molecules (e.g. antipsychotics and anxiolytics) did not alter mobility of Gsα [12].

## Conclusions

Taken together, these observations demonstrate that the increased liberation of Gsα from the TX-100 insoluble lipid raft membrane domains is unique to antidepressant drugs. Additionally, this result appears to be Gsα specific as Giα does not have the same response. Previous studies have demonstrated this outcome as well [7, 8, 25]. These findings are supported by evidence suggesting that antidepressant drugs concentrate in raft-like plasma membrane domains [11], which may physically inhibit the localization of Gsα. Thus, it appears that one effect of antidepressants may be to exert their observed effects on cAMP signaling by liberating Gsα from TTX-100 resistant membrane domains, where it accumulates during the course of depression. Mood stabilizing drugs like lithium and valproate do not affect lipid raft membrane domains and do not increase association of Gsα with adenylyl cyclase. The alteration in the membrane localization of Gsα may one day prove to be one of a number of useful biomarkers for human depression and response to antidepressant therapy.

## Methods

### Cell culture and drug treatment

C6 cells in 150 cm^2^ flasks were cultured in Dulbecco’s modified Eagle’s medium, 4.5 g of glucose/l, 10% newborn calf serum (Hyclone Laboratories, Logan, UT, USA), 100 mg/ml penicillin and streptomycin at 37°C in humidified 5% CO_2_ atmosphere. The cells were treated with 10 µM escitalopram (gift from Lundbeck, Copenhagen, Denmark), 3 mM Lithium chloride (Sigma-Aldrich, St. Louis, MO, USA), 300 µM sodium valproic acid (Sigma-Aldrich, St. Louis, MO, USA), dissolved in water. Cells were treated for 3 days as 3 day treatment in culture has given results similar to chronic in vivo treatment for 3 weeks in rats [7]. The culture media and drug were changed daily. There was no apparent change in morphology of cells during the period of exposure to antidepressants.

### Cell membrane, lipid raft, and detergent extract preparation

After treatment, cells were washed and harvested in ice-cold Phosphate-Buffered Saline (Mediatech Inc.). TX-100 insoluble membrane fractions were prepared as described by Li et al. [24], with slight modification [4]. In brief, two flasks of C6 cells were scraped into 0.75 ml of HEPES buffer (10 mM HEPES, pH 7.5, 150 mM NaCl, 1 mM DTT, and protease inhibitors) containing 1% TX-100. Cells were homogenized with 10 strokes of a Potter–Elvehjem homogenizer. The homogenate was then mixed with an equal volume of 80% sucrose prepared in HEPES buffer to form 40% sucrose and loaded at the bottom of an ultracentrifuge tube. A step gradient was generated by sequentially layering 30, 15, and 5% sucrose over the homogenate. Gradients were centrifuged at 2,00,000*g* for 20 h in an SW55 rotor (Beckman, Palo Alto, CA, USA). Two or three opaque bands were confined between the 15 and 30% sucrose layers. These bands were removed from the tube, diluted threefold with HEPES buffer, and pelleted in a microcentrifuge at 16,000*g*. The pellet was resuspended in HEPES buffer and subsequently analyzed by immunoblotting.

### SDS-PAGE and western blotting

Samples were assayed for protein via a bicinchoninic acid assay (Pierce Research, Rockford, IL, USA) and equal quantities were loaded onto Stain-Free acrylamide gel for SDS-PAGE (Bio-Rad, Hercules, CA, USA). Gels were transferred to Immobilon-P PVDF membranes (EMD Millipore, Billerica, MA, USA) for western blotting. The membranes were blocked with 5% nonfat dry milk diluted in TBS-T (10 mM Tris–HCl, 159 mM NaCl, and 0.1% Tween 20, pH 7.4) for 1 h. Following the blocking step, membranes were washed with Tris-buffered saline/Tween 20 and then incubated with an anti-Gsα monoclonal antibody (NeuroMab clone N192/12, Davis, CA, USA, catalog # 75-211, RRID #AB_2315846), anti-Gsα polyclonal antibody (EMD Millipore, Billerica, MA, USA, catalog # 06-237, RRID # AB_310078), or anti-Giα polyclonal antibody (Thermo Scientific, Rockford IL, USA, catalog # PA1-1000, RRID # AB_2232440) overnight at 4°C. Membranes were washed with TBS-T and incubated with a secondary antibody [HRP-linked anti-mouse antibody IgG F(ab′)2 or HRP-linked anti-rabbit antibody IgG F(ab′)2] (Jackson ImmunoResearch, West Grove, PA, USA, catalog # 115-036-072 for mouse, RRID # AB_2338525 and catalog # 111-036-047 for rabbit, RRID #AB_2337945) for 1 h at room temperature, washed, and developed using ECL Luminata Forte chemiluminescent reagent (Millipore, Billerica, MA, USA). Blots were imaged using Chemidoc computerized densitometer (Bio-Rad, Hercules, CA, USA) and quantified by ImageLab 3.0 software (Bio-Rad, Hercules, CA, USA). In all experiments, the original gels are visualized using BioRad’s Stainfree technology to verify protein loading.

### Fluorescence recovery after photobleaching (FRAP)

C6 glioma cells were transfected with either GFP-Gsα or GFP-Giα_1_ and cells stably expressing the fluorescent construct were selected with G418 followed by fluorescence activated cell sorting and isolation of clones [12]. Cells were plated on glass microscopy dishes and treated with lithium chloride (3 mM), valproic acid (300 μM), escitalopram (10 μM), or escitalopram plus lithium combined (same doses as individual treatments with these compounds) for 3 days. On the day of imaging, drug was washed out for 1 h prior to imaging and media was replaced with low serum (2.5% NCS) phenol red-free DMEM to reduce background fluorescence. Temperature was maintained at 37°C using a heated stage assembly during imaging, which utilized a Zeiss LSM 710 at 512 × 512 resolution using an open pinhole to maximize signal but minimizing photobleaching. 150 data points approximately 300 ms apart, including 10 pre-bleach values, were measured for each cell. Zeiss Zen software was used to calculate FRAP recovery half-time utilizing a one-phase association fit, correcting for total photobleaching of the analyzed regions.

### Statistical analysis

All of the experiments were performed at least three times. Data were analyzed for statistical significance using a one-way ANOVA followed by Tukey test for post hoc multiple comparisons of means. Values of p < 0.05 were taken to indicate significance.
